# Biomonitoring Data for Assessing Aflatoxins and Ochratoxin A Exposure by Italian Feedstuffs Workers

**DOI:** 10.3390/toxins11060351

**Published:** 2019-06-18

**Authors:** Barbara De Santis, Francesca Debegnach, Elisa Sonego, Gianmarco Mazzilli, Francesca Buiarelli, Fulvio Ferri, Paolo Giorgi Rossi, Giorgia Collini, Carlo Brera

**Affiliations:** 1 Reparto di Sicurezza Chimica degli Alimenti – Istituto Superiore di Sanità, 00161 Rome, Italy; francesca.debegnach@iss.it (F.D.); mazzilligianmarco@gmail.com (G.M.); carlo.brera@iss.it (C.B.); 2 Dipartimento di Chimica – Università “Sapienza”, 00185 Rome, Italy; elison.mail@gmail.com (E.S.); francesca.buiarelli@uniroma1.it (F.B.); 3 Servizio Prevenzione Sicurezza Ambienti di Lavoro, SPSAL – AUSL, 42122 Reggio Emilia, Italy; fulvio.ferri@gmail.com; 4 Servizio Epidemiologia, AUSL – IRCSS di Reggio Emilia, 42122 Reggio Emilia, Italy; paolo.giorgirossi@ausl.re.it (P.G.R.); giorgia.collini@ausl.re.it (G.C.)

**Keywords:** mycotoxins, biomonitoring, aflatoxin B1, ochratoxin A, occupational exposure

## Abstract

Mycotoxins exposure by inhalation and/or dermal contact is possible in different branches of industry especially where heavily dusty settings are present and the handling of dusty commodities is performed. This study aims to explore the validity of the biomonitoring as a tool to investigate the intake of mycotoxins in a population of workers operating in an Italian feed plant. Serum samples were collected for the determination of aflatoxins B1 (AFB1), AFB1-Lysine adduct and ochratoxin A (OTA). A method based on liquid–liquid extraction coupled with high resolution mass spectrometry determination was developed and fully validated. For AFB1, a high number of non-detected samples (90%) was found and no statistical difference was observed comparing workers and control group. None of the analyzed samples showed the presence of AFB1-Lysine adduct. For OTA, the 100% of the analyzed samples was positive with a 33% of the samples showing a concentration higher than the limit of quantification (LOQ), but no statistical difference was highlighted between the average levels of exposed and control groups. In conclusion, the presence of AFB1 and OTA in serum cannot be attributable to occupational exposure.

## 1. Introduction

Microscopic filamentous fungi can develop on food commodities of plant origin (maize, wheat, etc.) and in some cases on foods of animal origin (meat products, raw ham, sausages, etc.) [1]. In suitable environmental conditions, these molds can produce chemical toxic compounds, known as mycotoxins, via their secondary metabolism [2]. Molecular structures of mycotoxins vary widely and, consequently, these xenobiotics may be classified according to their action on different target organs as hepatotoxins, nephrotoxins, neurotoxins, immunotoxins, or according to their toxicological effects as carcinogenic, genotoxic, mutagen, teratogen [3].

To date, more than 500 mycotoxins are known to the scientific community, not all of them have a proper chemical and toxicological characterization. Researchers have been focusing their interest especially to a limited number of compounds, among which aflatoxins and ochratoxin A have a leading role due to their toxicity, distribution and occurrence. Aflatoxin B1 (AFB1) is considered the most toxic mycotoxin. Due to its carcinogenicity and genotoxicity the International Agency for Research on Cancer (IARC) [4] has classified it in the group 1A. Ochratoxin A (OTA) is a worldwide spread mycotoxin occurring in a wide range of products [5], it is considered a possible risk factor for its adverse renal effects in humans (based on renal tumors in male rats [6,7]), and it was classified in the group 2B by IARC [4]. The AFB1 metabolism is predominantly carried out in liver; it is mediated by the activity of the cytochrome P450 system and by the enzymatic action of NADPH reductase [8]. Among all the metabolites, the exo-epoxide (AFBO) quickly reacts to form DNA, RNA, and proteins adducts, which are responsible of the AFB1 toxicity. Aflatoxin B1 and aflatoxin B1-Lysine adduct (AFB1-Lys) in proteolytic digests of serum have been extensively used to assess the exposure of individuals in biomonitoring studies [9,10,11,12]. Other hydroxylated metabolites, namely aflatoxin M1, aflatoxin P1, aflatoxin Q1 and aflatoxicol, have different binding potential with DNA, and are mainly excreted in faeces and urines [8]. The metabolism of OTA in animals and humans includes hydrolysis, hydroxylation, lactone opening and conjugation, therefore different metabolites can occur [13]. Ochratoxin A shows a strong affinity with plasmatic proteins and due to its bond with serum albumin, the parent compound has been suggested as a parameter in biomonitoring for the assessment of exposure [6].

The most common route of exposure to mycotoxins is ingestion through the diet, either by direct exposure (through the consumption of agricultural food products directly contaminated), or by indirect exposure through the consumption of food derived from animals fed with contaminated feedstuffs. In addition to food diet, humans and animals can also be exposed to mycotoxins by inhalation of contaminated dusts in indoor settings where respirable particles below 1.0 micron may be present as particulate material that can act as carriers for toxins [14]. Transdermal studies have proved that mycotoxins can penetrate and also contribute to the health risk and the kinetics measured of AFB1, OTA, fumonisin B1, citrinin, zearalenone and T-2 toxin, showed OTA to have the highest permeation while AFB1 the lower permeability rates [15]. Thus, in a more comprehensive environmental exposure assessment, inhalation and/or dermal routes should be also considered as they may be observed in certain agricultural environments (cereal repositories, feed plants or warehouses, for example), in waste management sectors or in mold-contaminated indoor environments [16,17]. Occupational contexts and related biomonitoring studies have been extensively reviewed by Viegas [16]. Exposure by inhalation and/or dermal contact is known in different branches of industry especially where heavily dusty settings are present and the handling of dusty commodities (e.g., grains, spices or coffee) is performed [18], values being in the range of 1 and 189 ng/mL for free AFB1 and 0.01 and 100 pg/mg for AFB1-albumin [8]. Several epidemiological studies reported a possible role of aflatoxins as an occupational risk factor. McLaughlin [19] registered an increased risk of primary liver cancer in grain mills workers in Sweden in view of the potential presence of aflatoxins. Olsen [20], in a study of cancer risk among male employees at livestock feed processing companies, observed elevated risks for liver cancer and for cancers of the biliary tract, and judged the exposure to aflatoxins in the imported crops to be the most probable explanation for these findings. However, a matter of fact is that mycotoxins frequently occur in agricultural products. A Biomin survey on mycotoxins in foodstuff and feed (corn, soybean meal, wheat, dried distillers’ grains with soluble and finished feed samples) collected worldwide occurrence during 2009–2011 and showed that 33% and 28% of the samples were positive to AFB1 and OTA, respectively [21]. The outputs of biomonitoring studies conducted worldwide point out an issue of human exposure and where environmental conditions are favorable, mycotoxins can represent a risk also in occupational settings.

Due to their severe toxicological repercussions, the exposure to mycotoxins must be characterized by an accurate evaluation. Commonly, the classical procedure envisages the assessment of the exposure, either by a probabilistic or deterministic approach, through the diet by combining food intakes with the average contamination occurrence data. More recently, a parallel approach is emerging by measuring the presence of a biomarker, being the parent mycotoxin and/or its metabolite, in biological fluids. In both exposure assessments, there is an intrinsic number of uncertainties that must be taken into due account. From one hand, the classical approach may lack of representativeness in the intake assessment because of (i) the sampling, (ii) the managing of the censored occurrence data, (iii) the lack of disaggregated consumption data. On the other hand, to derive an exposure value from the measured biomarker, information about the in vivo metabolism, the toxicokinetics and toxicodynamics associated to the intake of the parent mycotoxins have to be known. Thus, there is still a certain approximation in the outputs mainly due to the high variability of the assessment and the related uncertainty, indicating that more research is needed for refinement and for overcome all knowledge gaps.

In recent times, the trend of analytical methods for mycotoxins or related metabolites is more often moving towards the multi-target methods coupled with mass spectrometry determination techniques. The target compounds are accurately quantified or semiquantitatively screened with validated methods with the support of guidance documents for the identification criteria [22]. In this analytical context, biomarkers (either parent compounds and/or metabolites) represent a rather new field, where the identification and the validation of biomarkers of exposure is still a challenge. A promising prospect is given by the application of recent high-resolution mass spectrometry (HRMS) techniques improving the accuracy of the analyses and opening the possibility to increase the number of explored molecules, thanks to the HR full scan recording application [23].

This study aims to explore the validity of the biomonitoring studies as a tool to investigate the intake of mycotoxins in a population of workers operating in workplaces, where the dusty settings trigger the inhalation and dermal contact of contaminated dust that may represent a risk of the exposure scenario.

With the objective to produce accurate exposure data and perform exposure assessment of these population groups, the study was conducted during winter 2017 on two groups: an exposed group of personnel working in an Italian feed plant, and a control group of non-exposed workers of administrative employees working in the same feed plant. Following the sampling plan defined, serum and urine samples were collected during two working days (Monday and Friday) for the determination of aflatoxins and OTA. Urine samples were scrutinized for short term exposure assessment (data not shown), and serum samples to assess the long-term exposure.

A method based on liquid–liquid extraction was developed and fully validated. The determination of the selected analytes in serum was performed by a high-resolution mass spectrometry (LC-HRMS) technique resulting in a suitable quantitative methodology with low limit of quantification (LOQ) to be used in biomonitoring context. Moreover, due to the unavailability of commercial standard of AFB1-Lys, the adduct was synthetized and used for the method set up and for qualitative analysis (presence/absence) in the collected samples.

## 2. Results and Discussion

### 2.1. Sample Preparation Optimization

Serum is a complex matrix and as a first experiment, a purification with a multi-mycotoxin immunoaffinity column (IAC) was applied. Commercially available IAC with specific antibodies for AFs and OTA (Aflaochra prep, R-Biopharm, Darmstadt, Germany) were tested, but the experiments gave unacceptable recoveries for OTA (R_E_ < 40%), suggesting that a pre-treatment of the sample is required prior IAC purification. However, taking into consideration the time of a single analysis, it was preferred to move to a liquid–liquid extraction strategy, being faster and cheaper than IAC and allowing retrospective analysis when a HRMS full scan is registered [24]. The extraction step was optimized starting from the method proposed by De Santis et al. [25]. Different mixtures were tested, namely ethyl acetate, ethyl acetate with 1% formic acid [25,26,27] and ethyl acetate added with a solution MgCl_2_ 0.1 M and HCl 0.05 M [28]. The results obtained are reported in Table 1 as apparent recovery.

The extraction with acidic solutions improved OTA extraction more than 10%, with no significant changes for AFB1, thus the ethyl acetate 1% formic acid was chosen for less use of reagent and solvent volume.

Since the hydrophilic metabolite AFB1-Lys wasn’t extracted in this mixture, a salting out step with a QuEChERS was applied to the serum residue. Three different solvents were tested for this second step, ethyl acetate, 1% formic acid ethyl acetate and acetonitrile (ACN). AFB1-Lys was extracted only with ACN; moreover, in none of the solvent tested AFB1 and OTA were still present, confirming the efficiency of the first extraction step.

Initially, the two organic fractions (from the first extraction and after the QuEChERS step) were collected in the same vial, but the solution obtained was cloudy also after centrifugation and filtration. Therefore, it was decided to collect and analyze separately the two fractions also reducing the number of interferences and matrix effect during LC-HRMS determination. The first extract was used for the quantitative determination of AFB1 and OTA, whilst the second was used for AFB1-Lys presence/absence evaluation.

After evaporation, samples were re-dissolved in MeOH:H_2_O 10:90 *v:v*, and 250 and 500 µL volume were tested. The higher dilution was preferred since it gave better results in terms of SSE without significance with respect to LOD values losses.

To reduce the high lipid fraction content of serum, the samples were shaken with n-hexane after pronase digestion prior to extraction. This step was not affecting the method performances.

### 2.2. Method Performance

#### 2.2.1. Linearity

Linearity was checked in the working range by the lack-of-fit test based on the analysis of variance (F test with *p*_value_ < 0.05) and the plot of the residual values randomly distributed around zero, confirming the linearity. During routine analytical sessions a *R*^2^ > 0.990 was set as a criterion for calibration curve acceptability. During analytical sessions *R*^2^ were in the range 0.9940–0.9992 for AFB1 and 0.9948–0.9995 for OTA (RSD, relative standard deviation was 0.2% for both mycotoxins) meeting the requirement.

#### 2.2.2. Apparent Recovery, Matrix Effect, and Extraction Recovery

The method allows the quantitative determination of AFB1 and OTA. The validation was conducted on 5 different levels of contamination, including the limit of quantification (LOQ). The results obtained during the validation process are reported in Table 2. The presence/absence of the AFB1-Lys adduct was considered during method set up, but excluded from the validation process.

While RSD_r_ (relative standard deviations of repeatability) complies with requirements established by this laboratory (≤ 20%) based on different reference standards [29,30], R_A_ percentages are relatively low, even if, considering reference standards for AFB1 at these low levels and the complexity of the analyzed matrix [29,30], reasonably acceptable.

Notwithstanding the liquid–liquid extraction is considered to allow a limited purification of the sample, the matrix effect experienced can be considered negligible, especially for OTA, for which SSE falls in the range 90–120% where it is reported to be negligible [31].

### 2.3. Application to Workers Serum Samples

The validated method was applied to the workers’ serum samples.

The LOQ levels were assessed during validation, and more specifically, the LOQ corresponded to the first validated contamination level, consistently with the established criteria of precision and trueness. The limit of detection (LOD) was estimated case by case in the analyzed samples: values lower than LOQ were reported in the dataset as positive samples provided that the identification criteria were met. According to the criteria reported in the DG SANTE guidance document on identification of mycotoxins in food and feed [22], the retention time (RT) of the analyte in the sample extract should correspond to the RT average of the calibration standards measured in the same sequence with a tolerance of ±0.1 min. Moreover, for the ^13^C-isotopically labeled analogue of the analyte (internal standard) added to the sample extract, the RT of the analyte should correspond to that of its labeled internal standard with a tolerance of ±0.05 min. In HRMS analysis, the identification is based on observation of the molecular ion (or, if not available, adducts) and at least one fragment that is specific for the selected analyte. All the mentioned criteria were met during validation and sample analyses. Due to the variation in matrix composition, matrix effect tolerances were also evaluated. An acceptance criterion was set with respect to the R_A_ values calculated for each analyzed sample as the ratio between the area of the labeled ISTD in the sample and the mean area of the labeled ISTD in pure solvent standard solutions used for the calibration curves. The samples with R_A_ < 40% were excluded.

The remaining results are to be considered as left censored data and for LB (lower bound) and UB (upper bound) mean calculation a substitution method was applied. In particular, for LB and UB calculation, zero and LOQ values were used respectively. The results obtained are summarized in Table 3.

A statistical analysis was carried out to describe the analytical results dataset. The hypothesis of normality distribution (Shapiro–Wilk test) was refused, thus all data was treated by a non-parametric statistics. Differences between concentration levels of mycotoxins in exposed and non-exposed groups were explored by a Wilcoxon rank-sum test. To assess the correlation between mycotoxin levels, a Spearman’s rank correlation coefficient (or Spearman’s rho) was used. All tests were conducted with a level of significance of 5%. Analyses were conducted by means of STATA14 software (Stata/IC 14.0, Copyright 1985–2015 StataCorp LP).

Aflatoxin B1 was detected in 6 samples from the workers group (Monday/Friday deliveries) ranging from 12.2 to 947.4 pg/mL_serum_, while only one positive sample was measured in the controls (19.7 pg/mL_serum_). However, due to the high number of non-detected samples (90.3% and 98.1% in the exposed and control group, respectively), no statistical differences were observed either comparing AFB1 Monday and Friday values in each group (exposed and non-exposed workers), or comparing values between exposed and non-exposed workers group (Monday and Friday merged) (Wilcoxon rank-sum test). None of the analysed samples showed the presence of AFB1-Lys adduct, even if it should be reminded that it was not possible to define the concentration level of the synthetized product; therefore, the adduct absence may be due to high LOQ value of the method.

In consideration of the high toxicity of AFB1 the positive results were also compared with available data. Only data referring to European population were considered for evaluating the occupational contribution with respect to the AFB1 exposure via the diet. The AFB1 ranges, detected in different occupational settings, are reported in Table 4. The amount of AFB1 measured in the present study is significantly below the reported results for other occupational setting. Moreover, if the ELISA method used in the other studies is applied to our analyses no positive samples would be found in both group workers and controls.

Considering the different legislation levels for AFB1 in food and feed (in the range 0.1–8 µg/kg for food and 0.5–20 µg/kg for feed) risky contaminated environments are more probable from the feed sector; occurrence of AFB1 in serum is low, thus, indicating that evidence for widespread exposure is lacking. Nevertheless, low level of exposure in a small number of persons cannot be excluded.

In conclusion, the low incidence of aflatoxins in serum cannot be attributable to occupational exposure and reveals a safe scenario, also for dietary exposure to AFB1.

For OTA the 100% of the analysed samples was positive with a 33% of the samples showing a concentration higher than the LOQ.

After scrutiny of the samples within and between the groups, it is possible to affirm that no statistical differences were highlighted between the average levels of exposed and control groups and no statistical differences were highlighted between Friday and Monday OTA levels, confirming that, in this context (corn-based feedstuffs company), this mycotoxin is not relevant for workers’ exposure. For OTA, only dietary exposure was suggested, and data obtained was used to estimate the individual daily intakes (EDIs). The EDIs were calculated on the basis of serum toxin levels using the Klaassen equation:

(1) $$k_{0}=\frac{Cl_{p}\times C_{p}}{A}$$

where *k*_0_ is the daily intake (ng/kg bw/day); *C_lp_* is the plasma clearance (mL/kg bw/day); *C_p_* is the serum concentration of OTA (ng/mL_serum_) and *A* is the bioavailability of the toxin. In this study, calculations were made with *C_lp_* = 0.99 mL/kg·bw/day according to Schlatter et al. [36] and *A* = 0.5 considering a 50% of OTA bioavailability [37]. For *C_p_* it was assumed that serum specimen concentration might be approximated to plasma concentrations, according to Palli et al. [38].

Obtained EDIs were compared to the reported OTA Tolerable Daily Intake (TDI) to evaluate the possible health concern due to this level of exposure. In particular, EFSA TDI of 17.14 ng/kg bw [6] was considered. The calculated EDI values were very low when compared with the EFSA TDI, with the highest value estimated being 12.77 ng/kg bw/day related to the Friday delivery of a volunteer from the control group. Also, the more recently Kuiper-Goodman et al. [39] re-evaluated TDI at 4 ng/kg·bw/day was considered. In this case, five subjects showed an EDI above that threshold value, three from the workers group, one sampled on Monday (5.70 ng/kg bw/day) and two on Friday (7.32 and 5.62 ng/kg bw/day), and two from controls group, from Monday (4.61 ng/kg bw/day) and Friday (12.77 ng/kg bw/day) deliveries. Moreover, the EDIs exceeding the 4 ng/kg bw/day, for the workers’ group on Monday and Friday, were from the same subjects (namely 5.70–5.62 and 7.32–3.9 ng/kg bw/day). In total, the 6% of the EDIs exceeded the re-evaluated TDI.

The estimated daily intakes were also compared with the Italian daily intake reported in the SCOOP Task “Assessment of dietary intake of OTA by the population of European Union Members States” elaborated in 2002 [40]. For this purpose, a single EDI mean value was calculated considering that no statistical differences were observed between workers and controls group and between Monday and Friday deliveries. The mean value of the volunteers enrolled in this study (1.19 ng/kg bw/day) overlaps with SCOOP Task value (1.16 ng/kg bw/day), confirming that the estimated exposure is mainly due to the diet.

In a previous work, published by Brera et al. [18] it is reported the OTA levels in serum collected from Italian volunteers working in cocoa, coffee and spices processing plants located in Tuscany, were lower than, but in line with, the discussed values, in this case the derived EDIs.

## 3. Conclusions

In the present study, a method has been set and validated inhouse for the determination of aflatoxin B1 and ochratoxin A in serum, also taking into consideration the presence of serum AFB1 adduct with lysine (AFB1-Lys). The method performances are encouraging since found acceptable and satisfactory in the context of a multi-toxin analytical method for serum being the data with HRMS spectrometry able to be used for retrospective screening. The LOD and LOQ values were very low, fitting with the bio-monitoring purpose that requires the determination of very limited toxins amount.

A future need is, undoubtedly, the organization of inter-laboratory studies with the aim of completing the validation process with a comparison study and harmonizing the analytical procedures to be applied for biological fluid analyses.

From the obtained results, it can be concluded that in the investigated occupational setting, the professional exposure seems to be not influential to the overall intake, being of the same order of magnitude of the control groups. Nevertheless, further studies are needed to also explore other settings where temperature and humidity conditions, together with indoors settings, for instance stables, might promote an occupational exposure via inhalation or skin contact.

## 4. Materials and Methods

### 4.1. Chemicals and Reagents

Chemicals and solvents used for sample preparation were ‘pro-analysis’ quality or better. LC-MS grade solvents, including water, methanol, ethyl acetate, acetonitrile and formic acid were purchased from Fisher Scientific (Milano, Italy). The following reagents were purchased from Sigma-Aldrich (Darmstadt, Germany): ammonium formate, protease from *Streptomyces griseus*, type XIV ≥3.5 units/mg, L-lysine (purity ≥98%). QuEChERS were from Waters (DisQuE, Waters, Milford, MA, USA).

The analytical reference standards of OTA were purchased as stock solutions (10 µg/mL in ACN) from Biopure (Tulln, Austria), crystalline powder of AFB1 from *Aspergillus flavus* (purity ≥98%) were purchased from Sigma-Aldrich (Darmstadt, Germany). The internal standards U-[^13^C_17_]-AFB1 (99.3% ^13^C) and U-[^13^C_20_]-OTA (99.2% ^13^C) were also purchased as ACN solution (0.5 µg/mL AFB1 and 10 µg/mL OTA) (Biopure, Tulln, Austria). Standard diluted solutions were prepared in ACN and stored at −20 °C in amber glass vials and used for the daily preparation of working solution.

Because of the absence of commercial AFB1-Lys standard at the moment of the study, the adduct was synthetized according to literature [41]. The collision energy value used for AFB1-Lys adduct was set referring to literature [41]. Due to the difficulties in assessing the concentration level of the synthetized adduct, it was used only for a qualitative evaluation of presence/absence in the collected serum samples.

### 4.2. Samples

The investigation was conducted in a large feedstuff plant located in Northern Italy (Reggio Emilia) that produces every year about 540,000 metric tons of feedstuffs (e.g., flour, compost and pellet), nearly 100,000 metric tons derived from maize. The plant management of the company agreed to participate according to the criteria and principles set by Italian legislation on workers’ health and safety and the study on human samples was also agreed with trade union representatives and the competent medical team. The workers were informed and gave formal consent. Samples were collected from two groups of volunteers, the exposed group, corresponding to all workers in direct contact with some risky activities, employees working in dusty plant areas, such as the downloading of the raw material, its handling and the cleaning procedures, and the control group corresponding to people working in the same company, but only involved in administrative activities, to be considered not risky for the absence of contaminated environmental dusts.

The collection of human serum samples was carried out in the morning on the work site by a physician and a nurse. A total of 61 male volunteers were enrolled. Blood samples were collected on Monday and Friday morning of the same working week from 32 exposed workers and 29 non-exposed workers as control group. Monday was chosen since it reflects a situation characterized by a preceding two-days washing period and Friday was selected with the aim to verify the possible accumulation of mycotoxins intake over the week of sampling. Blood samples were collected in 10-mL cryogenic tubes and immediately transported in refrigerated boxes at 0 °C to the analytical laboratory and stored at −20 °C until analysis.

### 4.3. Sample Preparation

**Figure 1:**
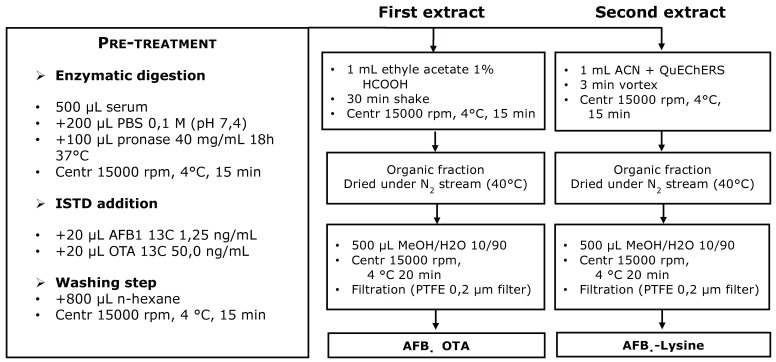
Scheme of the sample preparation steps, including labelled standard addition for quantification purpose.

Serum samples were allowed to reach room temperature. Five hundred µL were diluted with 200 µL of phosphate-buffered saline PBS (pH = 7.4) and mixed with pronase solution (100 µL, 40 mg/mL) and incubated at 37 °C for 18 h. After enzymatic treatment with pronase, labeled internal standard solutions were added (20 µL U-[^13^C_17_]-AFB1 5 ng/mL; 20 µL U-[^13^C_20_]-OTA 5 ng/mL). Serum sample was shaken for 5 min with 800 µL of n-hexane and centrifuged at 15,000 rpm for 15 min at 4 °C. Aqueous fraction was transferred with a syringe in a 2 mL Eppendorf tube and the sample was extracted with 1 mL of acidified ethyl acetate (1% formic acid) by shaking for 30 min. The sample was centrifuged at 15,000 rpm for 15 min at 4 °C and the supernatant transferred in a collection amber vial. One mL of ACN was then added to the serum residue, sample is vortexed and mixed with 300 mg of QuEChERS (DisQuE, Waters, Milford, MA, USA). The sample was centrifuged at 15,000 rpm for 15 min at 4 °C and the organic layer transferred in a separate collection vial. Both collected organic phases were evaporated to dryness, reconstituted in 500 µL of MeOH:H_2_O 10:90 *v:v* and 20 µL injected into UHPLC-HRMS system for the LC-MS/MS analysis. The described analytical procedure is outlined in Figure 1.

### 4.4. LC-HRMS Analysis

The optimization of the MS parameters was performed by infusion of standard mycotoxin solutions in presence of the mobile phase mixtures. The electrospray ionization (ESI) source parameters and the collision energy values were optimized to maximize signal and the fragmentation of precursor ions. The mobile phases were methanol and water (10:90), both added with formic acid and ammonium formate to help the production of protonated precursor ions.

The time of the chromatographic run was optimized to perform analytes separation, complete elution of the matrix components and to reduce interferences and signal suppression or enhancement (SSE).

The determination was performed with high-resolution mass spectrometer Q-Exactive Orbitrap in Full Scan/Data Dependent (full MS/dd-MS2) acquisition mode.

Mycotoxins quantification was performed with the internal standard (ISTD) approach using the U-[^13^C_17_]-AFB1 and U-[^13^C_20_]-OTA standards. The calibration curves were prepared in MeOH:H_2_O 10:90 v:v and obtained by plotting the ratio (standard area/^13^C area) versus the concentration expressed in pg/mL_serum_ for six concentration levels (namely 2.5, 5.0, 12.5, 25.0, 37.5 and 50.0 pg/mL for AFB1, and 250, 500, 1250, 2500, 3750 and 5000 pg/mL for OTA). Standard calibration solutions were daily prepared. For the sample analysis, ISTD was added to the sample at the very beginning, before sample extraction, in order to take into account the total recovery (R_A_) due to extraction recovery (R_E_) and SSE (Signal Suppression Enhancement) contributions.

Chromatographic separation was performed using UHPLC Dionex UltiMate 3000 (Thermo Scientific, San Jose, CA, USA) with Waters RP column Acquity BEH C18 (1.7 µm, 100 × 2.1 mm, Milford, MA, USA). The flow rate was set at 0.3 mL/min and the column temperature at 40 °C. The mobile phases A and B were water and methanol containing 0.002% formic acid and 2 mM ammonium formate. The following step gradient was used: 10% B increase to 99% in 10 min, keep isocratic at 99% B for 4 min, from 14 to 14.6 min return to 90% B, and finally re-equilibrate the column at 10% B for 2.4 min. The injection volume was set at 20 µL. High-resolution MS (HRMS) analysis was performed using Q-Exactive Orbitrap equipped with HESI source (Thermo Scientific, San Jose, CA, USA). The following ESI (+) parameters were used: source voltage 3.5 kV, capillary temperature 320 °C, auxiliary gas heater temperature 350 °C, sheath gas flow 40, S-lens RF level 75 and auxiliary gas flow 14. The MS acquisition was performed in Full Scan/Data Dependent (full MS/dd-MS^2^) for confirmatory purpose. In this acquisition mode the Q-Exactive Orbitrap automatically switch between full scan (mass range 100–700 *m*/*z*; automatic gain control target 1 × 10^6^ ions, and resolution of 70,000) and MS/MS acquisition, performing data-dependent scans (isolation window = 4.0 *m*/*z*; automatic gain control target 2 × 10^5^; and resolution 17,500). Precursor ions, selected by the quadrupole, are sent to the HCD collision cell; here they are fragmented to obtain ion spectra. Normalized collision energy (NCE) was set at 50 and 40 for AFB1 and OTA respectively and 37 eV collision energy (CE) is set for the AFB1-Lys adduct. Precursor ion, fragments, collision energy, and retention time used for the determination of the selected mycotoxins are reported in Table 5.

All analytical batches included analysis of appropriate extracts and solvent blanks, solvent calibration curves at the beginning, in the middle and at the end of the analytical batch, and injection of a calibration level every 10 sample injections to ensure LC–MS stability throughout the run. For data acquisition and processing Xcalibur software 4.0.27.19 was used. Mycotoxins were quantitated using the precursors ion signals which were extracted with ± 5 ppm window.

**Figure 2:**
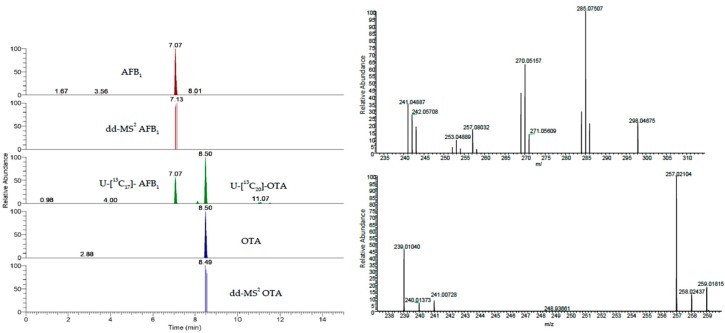
From top left, the extracted ion current of AFB1 and its data dependent, the extracted ion currents of the labelled internal standard of AFB1 and OTA and the extracted ion current of OTA and its data dependent. On the right side, the mass spectra of AFB1 (top) and OTA. The data are from the injection of a spiked serum sample (AFB1 25 pg/mLserum, OTA 2500 pg/mLserum).

Chromatograms and mass spectra of a spiked serum sample are reported in Figure 2 as an example.

### 4.5. Method Performance

#### 4.5.1. Linearity

Linearity of the method was evaluated from six points calibration curves injected in triplicate for three consecutive days, for each mycotoxin/matrix combination. The linearity was assessed by visual checking of the residual plot of response ratios (plotted in y-direction) versus the respective concentration levels (plotted in x-direction). The final estimated linearity model was verified using the lack-of-fit test (significance of the test with p_value_ below 0.05), to confirm that the selected regression and linearity were acceptable. Once visual checking of the residual and lack-of-fit test passed, the R squared coefficient was taken as a measure of linearity.

#### 4.5.2. LOD and LOQ Estimation

According to Wenzl [42], spiked blanks approach was used for LOD and LOQ assessment, by analyzing the spiked sample in ten replicates under repeatability conditions. The variability expressed as standard deviation obtained for the ten analyses of spiked blanks was used for the estimation of the critical value of LOD. Calculations were carried out according to Equations (2) and (3).

x_LOD_ = 3.9 × (s_y,b_/b) (2)

x_LOQ_ = 3.3 x_LOD_ (3)

The LOQ values, obtained with the theoretical calculation approaches shown in Table 3, were added to the validation levels and validated as described below.

#### 4.5.3. Apparent Recovery, Matrix Effect, and Extraction Recovery

Since no certified reference materials were available for mycotoxins in serum, the validation was performed by spiking experiments on pooled blank serum. Five different contamination levels including LOQ were used, namely 5.00, 7.00, 15.50, 25.00 and 50.00 pg/mL_serum_ for AFB1 and 500, 700, 1250, 2500 and 5000 pg/mL_serum_ for OTA. Due to the limited amount of blank matrix, validation experiments were conducted with the aim of minimizing the amount of sample needed. For this purpose, blank samples were spiked at the beginning of the analytical procedure, while isotopically labelled internal standards were added before injection into LC-HRMS system. The obtained data were used for R_A_ (ratio between the slopes of spiked sample and pure solvent), matrix effect (SSE, ratio between the ISTD mean area in sample extract and in pure solvent) and R_E_ (ratio between R_A_ and SSE) calculations. The apparent recovery was used for trueness evaluation, while precision was estimated in terms of intermediate precision as RSD_r_.

## Figures and Tables

**Table 1:** Recovery factor values (R_A_, %) and associated relative standard deviation (RSD, %) for the solvent mixtures tested for AFB1 and OTA extraction.

Solvent Mixture	R_A_ ± RSD	
	AFB1	OTA
Ethyl acetate	61 ± 17	52 ± 13
Ethyl acetate 1% formic acid	59 ± 12	65 ± 9
Ethyl acetate MgCl_2_ 0.1 M and HCl 0.05 M	60 ± 12	70 ± 15

**Table 2:** Method validation parameters.

Parameters	AFB1	OTA
LOD (pg/mL_serum_)	1.5	180.0
LOQ (pg/mL_serum_)	5.0	500.0
Working range (pg/mL_serum_)	5.0–50.0	500.0–5000.0
R_A_, %	55	61
R_E_, %	67	63
SSE, %	82	96
RSD_r_, %	11	9

**Table 3:** Results of AFB1 and OTA serum biomarkers of worker and control groups.

Exposed Workers Group	AFB1	OTA
**Monday and Friday; subjects (*n* = 63)**		
Excluded (n)	1	4
Positive ^a^ (n)	6	59
Positive (%)	9.7	100
Max ^b^ (pg/mL_serum_)	947.4	3700
Mean (LB-UB) (pg/mL_serum_)	24.2–28.7	-
Mean positive (pg/mL_serum_)	249.9	600
Median (pg/mL_serum_)	-	380 ^c^
**Monday; subjects (*n* = 32)**		
Excluded (n)	1	3
Positive ^a^ (n)	4	29
Positive (%)	12.9	100
Max ^b^ (pg/mL_serum_)	289.8	2880
Mean (LB-UB) (pg/mL_serum_)	16.7–21.0	-
Mean positive (pg/mL_serum_)	249.9	600
Median (pg/mL_serum_)	-	360
**Friday; subjects (*n* = 31)**		
Excluded (n)	-	1
Positive ^a^ (n)	2	30
Positive (%)	6.5	100
Max ^b^ (pg/mLs_serum_)	947.4	3700
Mean (LB-UB) (pg/mL_serum_)	31.7–36.4	-
Mean positive (pg/mL_serum_)	491.8	600
Median (pg/mL_serum_)	-	380
**Control Group**	**AFB1**	**OTA**
**Monday and Friday; subjects (*n* = 55)**		
Excluded (n)	3	5
Positive ^a^ (n)	1	50
Positive (%)	1.9	100
Max ^b^ (pg/mL_serum_)	19.7	6450
Mean (LB-UB) (pg/mL_serum_)	0.4–5.3	-
Mean positive (pg/mLs_serum_)	-	600
Median (pg/mL_serum_)	-	370
**Monday; subjects (*n* = 28)**		
Excluded (n)	1	2
Positive ^a^ (n)	0	26
Positive (%)	-	100
Max ^b^ (pg/mL_serum_)	-	2330
Mean (LB-UB) (pg/mL_serum_)	-	-
Mean positive (pg/mL_serum_)	-	530
Median (pg/mL_serum_)	-	450
**Friday; subjects (*n* = 27)**		
Excluded (n)	2	3
Positive ^a^ (n)	1	24
Positive (%)	4	100
Max ^b^ (pg/mL_serum_)	19.7	6450
Mean (LB-UB) (pg/mL_serum_)	0.8–5.6	-
Mean positive (pg/mL_serum_)	-	680
Median (pg/mL_serum_)	-	350

^a^ Positive: values above LOD; ^b^ Max: maximum value; ^c^ Value below the LOQ.

**Table 4:** Comparison of AFB1 levels in different European occupational settings.

Reference	Country	Occupational Setting	Sampling Details	Method, LOD/LOQ (ng/mL_serum_)	AFB1 Range (ng/mL_serum_)
Present study	Italy	Feed mill workers	32 workers, 29 controls	LC-HRMS, LOD = 0.0015	0.0122–0.947 0.0197 controls group
Viegas et al., 2013 [32]	Portugal	Swine husbandry	28 workers, 30 controls	ELISA, LOD = 1	<LOD-8.94 No AFB1 detected in the controls
Viegas et al., 2015 [33]	Portugal	Waste management	41 workers, 30 controls	ELISA, LOD = 1	2.5–25.9, No AFB1 detected in the controls
Viegas et al., 2016 [34]	Portugal	Poultry slaughterhouse	30 workers, 30 controls	ELISA, LOD = 1	1.06–4.03 No AFB1 detected in the controls
Ferri et al., 2017 [35]	Italy	Feed mill workers	29 workers, 30 controls	HPLC-FLD, LOD = 0.025	No AFB1 detected

**Table 5:** Precursor ion, fragments, collision energy and retention time used for the determination of the selected mycotoxins in serum samples.

Mycotoxin	Chemical Formula	Precursor Ion (*m*/*z*) [M+H]^+^	Fragment (*m*/*z*)	NCE	Retention Time (min)
AFB1	C_17_H_12_O_6_	313.07066	285.07571; 241.04952	50	7.07
U-[^13^C_17_]-AFB1	^13^C_17_H_12_O_6_	330.12770	-	-	7.07
AFB1-Lys	C_23_H_24_N_2_O_8_	457.16054	394.12782, 328.08112	37 (CE)	4.84
OTA	C_20_H_18_ClNO_6_	404.08954	257.02147; 239.01087	40	8.48
U-[^13^C_20_]-OTA	^13^C_20_H_18_ClNO_6_	424.15664	-	-	8.48
